# The Book World of Medicine and Science

**Published:** 1893-11-04

**Authors:** 


### The Book World of Medicine and Science.

Life of Sir Richard Burton. By Lady Burton. (Chapman and Hall.)

Readers of this biography will experience, it may safely be predicted, a variety of sensations on their way through its two closely printed volumes. Curiosity, amusement, breathless interest, incredulity, and regret all have their place, while admiration and disapproval jostle one another until it is hard to say which is uttermost. Perhaps on the whole the incredulity carries the day. We stumble too often into a dimly lighted region where gipsy prophecies and vulgar ghost stories link themselves to the latest development in hypnotism or zoo-electricity and psychical research. It is open to every one to tell stories of this nature, but this being a free country, plain people are still at liberty to say in plain terms that they do not see their way to believing them. The book is, in fact, painfully full of superstition, and for that reason can hardly be accepted as a definite life of the great linguist and explorer. It is written moreover in a controversial spirit which gives the impression of a perpetual jar, and more than once rouses the desire to have the other side of the case fairly stated. It cannot be denied, however, that the book contains the most valuable biographical materials, and that it can be read from one end to the other without danger of falling asleep. Dulness is its last fault, and everyone will find himself bound to read it, in order to find out what his neighbour dislikes in it. In many respects the most entertaining part is the autobiographical fragment taken down by Lady Burton from her husband's lips, and containing an account of his youth and the rather mixed education which his father's wandering habits threw in his way in the course of their travels. Among wild escapades in Naples in 1836 occurs the following graphic description of the cholera panic: "The shady side of the picture was the cholera. It caused a fearful destruction, and the newspapers owned to 1,300 a day, which meant 2,300. The much-abused king behaved like a gentleman. The people had determined that the cholera was poison, and doubtless many made use of the opportunity to get rid of husbands and wives and other inconvenient relationships; but when the mob proceeded to murder the doctors, and to gather in the market square with drawn knives, declaring that the Government had poisoned the provisions, the king himself drove up in a phaeton and jumped out of it entirely alone, told them to put up their ridiculous weapons, and to show him where the poisoned provisions were, and seating himself upon a bench, ate as much as his stomach would contain. Even the lazzarone were not proof against this heroism and viva'd, and cheered him to his heart's content." Burton and his brother were proof against fear in any form, and their feeling about cholera was one of simple curiosity. "We persuaded the Italian man-servant to assist us in a grand escapade. He had procured us the necessary dress, and when the dead carts passed round in the dead of the night, we went the rounds with them as some of the 'croque-morts.' The visits to the pauper-house where the silence lay in the rooms, were anything but pleasant, and still less the final disposal of the bodies. . . . Black and rigid they were thrown down the apertures like so much rubbish into the festering heap below, and the decay caused a kind of lambent blue flame about the sides of the pit."

**THE BOOK WORLD OF MEDICINE AND SCIENCE**—*continued.*

Transactions of the Sanitary Institute, Vol. xiii., 1892. (Edward Stanford.)

No better illustration of the varied and far-reaching interests grouped together under the general title of Public Health, or of the scope of the work done by the Sanitary Institute could be had, than a glance at the contents of the thirteenth volume of its transactions, which is now before us. This volume is chiefly occupied by work of the Portsmouth Congress and of five subsidiary conferences, and the numerous papers deal with the most important sanitary problems of the day, and must therefore prove of great value to all interested in maintaining or improving the health of the people. In every direction there is yet much to be done in this respect, and in the face of a threatened invasion of cholera, though we trust in our coast-inspection to keep out the danger, it behoves the public, individually as well as collectively, to make such preparations as will limit its spread should it obtain a footing in the country.

As a sound knowledge of sanitary subjects cannot be too widely disseminated, we will endeavour to briefly notice the principal papers with special reference to those of general interest. Before coming to the work of the Congress we notice two papers which form an interesting contrast of views on the treatment of house refuse. Mr. Jones, of the Ealing Local Board, ably advocates the use of the destructor, the construction and mode of using which he fully describes. He claims that the most improved furnaces of this class will effectually destroy the varied ingredients of house refuse and street sweepings most cheaply and without nuisance, while providing power for electric lighting, &c. Mr. Joseph Russell, on the other hand, recommends a system of utilisation of a striking character even in this age of bye-products. By an arrangement of screens, travelling tables, and fans to be seen at the works of the Refuse Disposal Company, Chelsea) a cart load of rubbish is sorted out in five minutes into no less than thirteen classes of material, each having a certain commercial value, and this almost entirely by machinery! The cost of such works would probably be considerable, and, in the light of recent bacteriology, it may be doubted whether some of the classes of material ought to be preserved, but every one must admire the ingenious process by which the sorting is accomplished. Dr. Louis Parkes deals with the deterioration of the air and water of London, and discusses the causes and cure of the fog nuisance, although his statements will not afford much comfort to inhabitants of the metropolis.

The inaugural address to the Congress was delivered by Sir Charles Cameron, M.D. (Dublin), who took for his subject the progress of sanitary matters during the Victorian era—the age of sanitation, as he happily expressed it. In a masterly manner he sketched the rise and progress of the main lines of sanitary legislation, indicating their results by statistics showing a steadily decreasing death-rate, especially with regard to infectious diseases. Finally, he pointed out the directions in which we are to look for advance, notably in the provision of better houses for the working classes at a rent sufficiently low to secure their occupation by the class most prone to propagate nfectious disease. We commend the paper to those who idesire to know what has been done during the last half century and what may be done in the future.

The Modern Hospital. (Quarterly Review, John Murray).

The last number of the "Quarterly Review" contains an article on the Modern Hospital, which is of great interest. The subject matter is based largely upon "Hospitals and Asylums of the World," but much other well-known literature on the subject has been thoroughly grasped by the writer. The subject is ably and thoughtfully treated, and the whole article abounds in evidence of sound knowledge and common sense, and we can heartily commend it to our readers' attention.

